# KNL1 Regulates Ferroptosis Resistance and Migration in Lung Adenocarcinoma Cells via AMPK-mTOR Signaling

**DOI:** 10.32604/or.2026.075191

**Published:** 2026-03-23

**Authors:** Yiran Dong, Jingyue Wang, Jiayang Chen, Liang Mo, Yong You

**Affiliations:** 1 Research Laboratory of Translational Medicine, Hengyang Medical College, University of South China, Hengyang, China; 2 Department of Thoracic Surgery, The First Affiliated Hospital, University of South China, Hengyang, China

**Keywords:** Kinetochore scaffold 1, ferroptosis, AMP-actived protein kinase-mammalian target of rapamycin signaling pathway, epithelial-mesenchymal transition, lung adenocarcinoma

## Abstract

**Background:**

Lung adenocarcinoma (LUAD), the most prevalent histological subtype of lung cancer, remains a leading cause of cancer-related mortality due to late diagnosis, metastasis, and therapy resistance. The aim of the study is to investigate the role of Kinetochore Scaffold 1 (KNL1) in promoting LUAD progression and its underlying molecular regulatory mechanisms.

**Methods:**

KNL1 mRNA expression levels across 33 cancer types were analyzed using bioinformatics analysis based on the TCGA database. Immunohistochemistry (IHC) was used to assess KNL1 expression in LUAD and normal tissues. Stable KNL1-knockdown and KNL1-overexpressing LUAD cell lines were established using lentiviral infection. Western blotting (WB) was used to measure epithelial-mesenchymal transition (EMT) markers and ferroptosis-related protein expression. Cell migration was evaluated via scratch wound healing assays. The thiobarbituric acid (TBA) method was employed for the detection of malondialdehyde. a fluorescent probe was utilized to determine ferrous ion content. WB determined the phosphorylation ratios of AMP-activated protein kinase (AMPK) and mammalian target of rapamycin (mTOR) proteins.

**Results:**

1. KNL1 was highly expressed in 31 cancer types, including LUAD. Kaplan-Meier curves showed significantly shorter median survival in patients with high KNL1 expression. IHC confirmed upregulated KNL1 expression in LUAD tissues. 2. KNL1 overexpression significantly promoted LUAD cell migration and increased mesenchymal marker expression, whereas KNL1 knockdown exerted opposite effects. 3. KNL1 overexpression significantly reduced MDA content and Fe^2+^ levels in RSL3-treated LUAD cells while increasing the expression of key ferroptosis defense proteins; conversely, it markedly increased the accumulation of MDA and Fe^2+^ and downregulated these proteins. KNL1 overexpression significantly increased phosphorylated AMPK (p-AMPK) expression but decreased phosphorylated mTOR (p-mTOR) expression in RSL3-treated LUAD cells; conversely, it inhibited p-AMPK expression and activated p-mTOR.

**Conclusion:**

KNL1 promotes lung adenocarcinoma progression by suppressing ferroptosis through regulation of the AMPK-mTOR signaling pathway.

## 1 Introduction

Lung cancer represents a major global health burden,and is the leading cause of cancer-related mortality worldwide, with an estimated 1.8 million deaths in 2022—accounting for nearly one in five cancer deaths. Lung adenocarcinoma (LUAD) is the most common histological subtype [1]. Despite advances in targeted therapy and immunotherapy, the overall 5-year survival rate for lung cancer remains dismal, largely due to late-stage diagnosis resulting from the lack of effective early diagnostic biomarkers and the aggressive nature of the disease. LUAD patients often face a poor prognosis due to metastasis and therapeutic resistance, highlighting the urgent need to further elucidate the molecular drivers of tumor progression. Current research has focused on the critical role of epithelial-mesenchymal transition (EMT) and cell migration in tumor progression [2,3]; however, how these mechanisms interact with novel cell death pathways to sustain cancer cell survival remains to be fully explored. Notably, emerging forms of cell death, particularly ferroptosis, have been shown to be closely associated with tumor progression [4,5], yet the regulatory network of this process in LUAD has not been fully clarified.

Ferroptosis, an iron-dependent form of cell death driven by lipid peroxidation, has gained attention as a therapeutic target due to its potential to eliminate malignant cells [6,7]. However, its regulatory mechanisms in cancers such as LUAD remain unclear and are often influenced by complex signaling pathways. Key molecules such as glutathione peroxidase 4 (GPX4) [8] and ferroptosis suppressor proteins (e.g., Solute Carrier Family 7 Member 11, SLC7A11/xCT) are involved in regulating iron homeostasis; yet the upstream hub mediating escape from ferroptosis remains unidentified. This ambiguity extends to metabolic pathways. AMP-activated protein kinase (AMPK) and the mechanistic target of rapamycin (mTOR), as central regulators of cellular metabolism and survival, have controversial roles in ferroptosis [9–12]. Therefore, this study aims to identify whether there exists a key molecule that concurrently suppresses ferroptosis and drives malignant tumor progression.

Among the numerous prognosis-related genes identified in genomic studies of LUAD, overexpression of kinetochore scaffold 1 (KNL1) is significantly associated with shortened patient survival [13]. Preliminary evidence suggests that KNL1 influences mitotic function and may regulate EMT markers such as Slug and vimentin [14]; however, its impact on ferroptosis and overall tumor progression remains unknown. Given the promising clinical potential of ferroptosis inducers, elucidating whether KNL1 drives tumor progression by regulating ferroptosis is crucial for developing novel strategies to target KNL1 to overcome therapy resistance.

The AMPK-mTOR pathway is involved in metabolic and stress responses and may coordinate the regulation of ferroptosis. However, existing studies present contradictions: AMPK can either inhibit or promote ferroptosis depending on the context [15,16]. It is particularly noteworthy that how oncogenes such as KNL1 participate in this pathway in LUAD remains unclear, and direct evidence regarding KNL1’s regulation of AMPK-mTOR and its effect on cellular response to ferroptosis inducers is lacking.

In summary, this study investigates the regulatory role of KNL1 in LUAD metastasis and ferroptosis. The findings aim to provide a new basis for optimizing clinical treatment strategies for LUAD and promote the translational application of ferroptosis-targeted therapies.

## 2 Materials and Methods

### 2.1 Biological Materials and Reagents

#### 2.1.1 Cells and Main Reagents

The human lung adenocarcinoma cell line H1975(Catalog NO. SCSP-597) and A549(Catalog NO. SCSP-503) were purchased from the Cell Bank of the Chinese Academy of Sciences(Shanghai, China); RSL3 (MCE, HY-100218A, Monmouth Junction, NJ, USA); RPMI 1640 cell culture medium (Gibco, 11875093, Waltham, MA, USA); DMEM cell culture medium (Gibco, 11965092); fetal bovine serum (FBS, Gibco, A5669701); trypsin (Gibco, 25200072); Penicillin-Streptomycin(Gibco,15140122); KNL1 antibody (CST, # 26687, Danver, MA, USA, 1:1000); β-actin antibody (CST, #8457S, 1:1000); Slug antibody (CST, # 9585, 1:1000); Vimentin antibody (CST, # 5741, 1:1000); xCT antibody (CST, # 12691, 1:1000); GPX4 antibody (CST, # 52455, 1:1000); FTH1 antibody (CST, # 4393, 1:1000); AMPK antibody (CST, # 2532S, 1:1000); Phospho-AMPK antibody (CST, # 50081, 1:1000); mTOR antibody (CST, # 2983, 1:1000); Phospho-mTOR antibody (CST, # 2971, 1:1000); MDA assay kit (Beyotime Biotechnology, S0131S, Shanghai, China); Ferrous iron (Fe^2+^) detection probe (Dojindo Molecular Technologies, M489, Kumamoto, Japan); Absorbance Microplate Reader(Molecular Devices, CMax Plus, Shanghai, China); Fluorescence microscope (Thermo Fisher Scientific, EVOS M5000, Waltham, MA, USA).

#### 2.1.2 Clinical Specimens

A total of 90 LUAD patients who underwent radical resection at the First Affiliated Hospital of University of South China between January 2023 and August 2024 were enrolled in this validation cohort (see Table 1). This study was approved by the Ethics Committee of the First Affiliated Hospital of University of South China (Ethics Approval No: 2023220120001). Written informed consent was obtained from all participants prior to enrollment. The study was conducted in accordance with the principles of the Declaration of Helsinki. The Ethics Committee confirmed that the use of archival tissue specimens was covered under the general research consent obtained from patients during clinical care. Inclusion criteria: (1) Histologically confirmed primary LUAD based on 2021 WHO classification; (2) Treatment-naïve (no neoadjuvant therapy); (3) Complete clinicopathological records including smoking history, TNM stage, and differentiation grade. Exclusion criteria: (1) Inadequate clinical data ; (2) History of other malignancies within 5 years

**Table 1:** Association between KNL1 expression levels and clinicopathological characteristics in lung adenocarcinoma patients.

Characteristic	High Expression of KNL1	Low Expression of KNL1	*p* Value
N, n	61	29	
Age, median (IQR)	62.00 (53.00–68.00)	57.00 (52.00–62.00)	0.075
**Gender**			0.767
Male, n (%)	23 (37.70%)	10 (34.48%)	
female, n (%)	38 (62.30%)	19 (65.52%)	
**T**			0.785
1, n (%)	52 (85.25%)	25 (86.21%)	
2, n (%)	8 (13.11%)	4 (13.79%)	
3, n (%)	1 (1.64%)	0 (0.00%)	
**N**			0.817
0, n (%)	53 (86.89%)	24 (82.76%)	
1, n (%)	4 (6.56%)	2 (6.90%)	
2, n (%)	4 (6.56%)	3 (10.34%)	
**M**			<0.001
0, n (%)	61 (100.00%)	29 (100.00%)	
**Tumor staging**			0.870
I, n (%)	53 (86.89%)	24 (82.76%)	
II, n (%)	3 (4.92%)	2 (6.90%)	
III, n (%)	5 (8.20%)	3 (10.34%)	
**Tumor grading**			0.083
G1, n (%)	2 (3.28%)	3 (10.34%)	
G2, n (%)	36 (59.02%)	21 (72.41%)	
G3, n (%)	23 (37.70%)	5 (17.24%)	
**lymphatic metastasis**			0.452
No, n (%)	54 (88.52%)	24 (82.76%)	
Yes, n (%)	7 (11.48%)	5 (17.24%)	
**Spread through air spaces**			0.605
No, n (%)	28 (45.90%)	15 (51.72%)	
Yes, n (%)	33 (54.10%)	14 (48.28%)	

Note: Abb: KNL1: kinetochore scaffold 1.

### 2.2 Main Methods

#### 2.2.1 Bioinformatic Analysis

Differential analysis of KNL1 expression across various cancers was performed using the TCGA database (https://portal.gdc.cancer.gov, accessed on 1 January 2025) combined with the GTEx database (https://commonfund.nih.gov/GTEx, accessed on 1 January 2025). RNA-seq data and clinical data from 510 LUAD patients and 58 normal lung tissue samples were collected from the TCGA database. Based on the median expression level of KNL1 in LUAD patients, the tumor patients were divided into a high KNL1 expression group and a low KNL1 expression group. Differential expression of KNL1 between LUAD and adjacent normal lung tissues was analyzed using the Wilcoxon rank-sum test. All statistical analyses and visualizations were performed using R software version4.0.3(R Foundation for Statistical Computing, Vienna, Austria) with the following packages: ggplot2 (v3.3.5) for data visualization and dplyr (v1.0.7) for data manipulation.

#### 2.2.2 Immunohistochemistry (IHC)

Clinical tissue sample collection strictly followed standard procedures. Tissue microarrays were constructed and IHC experiments were performed by Shanghai Outdo Biotech Company Ltd. Staining was observed under a microscope, and scoring was performed by pathology experts. IHC scoring criteria: 0 = no staining; 1 = light yellow or light brown; 2 = yellow-brown; 3 = dark yellow or dark brown. Positive cell scoring: 1 = ≤10% positive cells; 2 = 11%–50% positive cells; 3 = 51%–80% positive cells; 4 = ≥80% positive cells. The final IHC score was calculated as: staining intensity score × positive cell percentage score, resulting in a score range of 0–12.

#### 2.2.3 Cell Culture and Grouping

A549 cells were cultured in DMEM medium supplemented with 10% FBS, and H1975 cells were cultured in RPMI 1640 medium supplemented with 10% FBS. Both were maintained in a 37°C, 5% CO_2_ incubator. A549 cells were divided into: Control group, RSL3 treatment group, and A549-OE-KNL1 group (overexpression). H1975 cells were divided into: Control group, RSL3 treatment group, and H1975-KD-KNL1 group (knockdown).

#### 2.2.4 Construction of Stable Cell Lines

Lentiviral vectors for knocking down and overexpressing human KNL1 were purchased from Tsingke Biotechnology. For knockdown, two shRNA sequences targeting human KNL1 were used: shKNL1-1 (5^′^-GGACATTACCAAGAGTCATAC-3^′^) and shKNL1-2 (5^′^-GGAAGTCACCGATTCCCATAC-3^′^). For overexpression, the CMV promoter was used in the pLV-CMV vector backbone. When cell confluence reached approximately 40%, cells were infected with KNL1-targeting lentivirus (for overexpression or knockdown; MOI = 30 for A549, MOI = 50 for H1975) in serum-free medium containing Polybrene (8 μg/mL), followed by incubation for 12 h. The medium was then replaced with complete medium containing 10% FBS. After 48 h, puromycin (1 μg/mL) was added to select for stably transfected cells. All experimental manipulations included matched empty vector controls processed in parallel under identical culture, transduction, and selection conditions to isolate KNL1-specific phenotypic effects. After 2–3 days of selection, cells were collected, lysed to extract total protein, and subjected to Western blotting to confirm the efficiency of KNL1 overexpression or knockdown.

#### 2.2.5 Western Blotting

Cells were collected and lysed with RIPA buffer to extract total protein. Protein concentration was determined using a BCA Protein Assay Kit (Beyotime Biotechnology, P0012). 15 µg of denatured total protein was loaded for 10% SDS-PAGE. After transfer and blocking at 4°C for 1 h, membranes were incubated with primary antibodies at 4°C overnight. Following three washes, membranes were incubated with secondary antibodies at room temperature for 2 h. Antibodies were used as follows: primary antibodies: KNL1, β-actin, Slug, Vimentin, xCT, GPX4, FTH1, AMPK, Phospho-AMPK, mTOR, Phospho-mTOR; secondary antibodies: Anti-mouse IgG (CST, # 9585, 1:10,000), Anti-rabbit IgG (CST, # 9585, 1:10,000). After another wash, bands were visualized using ECL chemiluminescent substrate (Beyotime Biotechnology, P0018AS) and imaged using an automated chemiluminescence imaging system(Tanon Science & Technology, Tanon5200, Shanghai, China). Gray values of protein bands were quantified using ImageJ version 1.8.0 (NIH, Bethesda, MD, USA), and the relative expression of target proteins was calculated using β-actin as the loading control. All experiments were performed with three independent biological replicates.

#### 2.2.6 Wound Healing Assay

Cell migration ability was assessed using a wound healing assay. 2000 cells were seeded into 6-well plates. After 24 h, when cells had adhered and reached a confluent monolayer, they were treated with 1 μg/mL mitomycin C (MCE, HY-13316) for 1 h before scratching and subsequent incubation in RPMI 1640 medium (for H1975 cells) or DMEM (for A549 cells; both from Gibco) without serum. Cell migration was observed and photographed at 0 h and 24 h post-scratch under an inverted microscope (Leica Microsystems, DMiL, Wetzlar, Germany) to analyze migration ability. Wound closure was quantified using ImageJ version 1.8.0 (NIH) by calculating [(area at 0 h–area at 24 h)/area at 0 h] × 100%

#### 2.2.7 MDA Detection

The malondialdehyde (MDA) content in cells, an indicator of lipid peroxidation, was measured using a Lipid Oxidation Assay Kit (Beyotime Biotechnology, S0131S). via the TBA method. Concurrently, total protein concentration in each sample was quantified using a BCA Protein Assay Kit (Beyotime Biotechnology, P0012) according to the manufacturer’s protocol. MDA levels were normalized to protein content (MDA/protein ratio) to account for cell number variations. Absorbance was measured at 530 nm for MDA detection and 562 nm for protein quantification. Notably, no positive control was included in the MDA assay as the manufacturer’s instructions for kit S0131S do not require this validation step. All experiments were performed with three independent biological replicates.

#### 2.2.8 Fe^*2+*^ Detection

Intracellular ferrous iron (Fe^2+^) content was detected using FerroOrange, a cell-permeable fluorescent probe that exhibits high selectivity for Fe^2+^ over Fe^3+^ and other biologically relevant metal ions. The detection principle is based on fluorescence enhancement upon specific binding to reduced iron (Fe^2+^), with minimal reactivity toward oxidized iron (Fe^3+^) under physiological conditions. Treated cells in 6-well plates were incubated with 1 mL of a 1 µmol/L working solution of the fluorescent probe. The plate was then placed in a 37°C, 5% CO_2_ incubator for 30 min. Fluorescence signals were acquired using an EVOS M5000 Imaging System (Thermo Fisher Scientific) with green excitation light (Ex: 532 nm/Em: ~580 nm). All Fe^2+^ detection experiments were performed with three independent biological replicates. No positive or negative controls (e.g., iron chelators or Fe^2+^ supplementation) were included, as the primary objective was comparative analysis of relative Fe^2+^ levels between experimental groups under identical treatment conditions, following established protocols for FerroOrange-based iron detection in cancer cell models

### 2.3 Statistical Analysis

Statistical analyses were performed using GraphPad Prism 10 (GraphPad Software, San Diego, CA, USA). All experiments included three independent biological replicates. Data normality was verified by the Shapiro-Wilk test; homogeneity of variance was assessed by Levene’s test. Normally distributed data are presented as mean ± SD and analyzed by: (1) unpaired Student’s *t*-test (two groups), or (2) one-way ANOVA with Tukey’s post-hoc test (≥3 groups). Non-normal data are expressed as median (IQR) and analyzed by the Kruskal-Wallis test with Dunn’s post-hoc test. Survival differences were evaluated by Kaplan-Meier analysis with the log-rank test. IHC data were analyzed using a paired *t*-test. *p* < 0.05 was considered significant.

## 3 Results

### 3.1 KNL1 Is Highly Expressed in Lung Adenocarcinoma and Associated with Poor Prognosis

**Figure 1:**
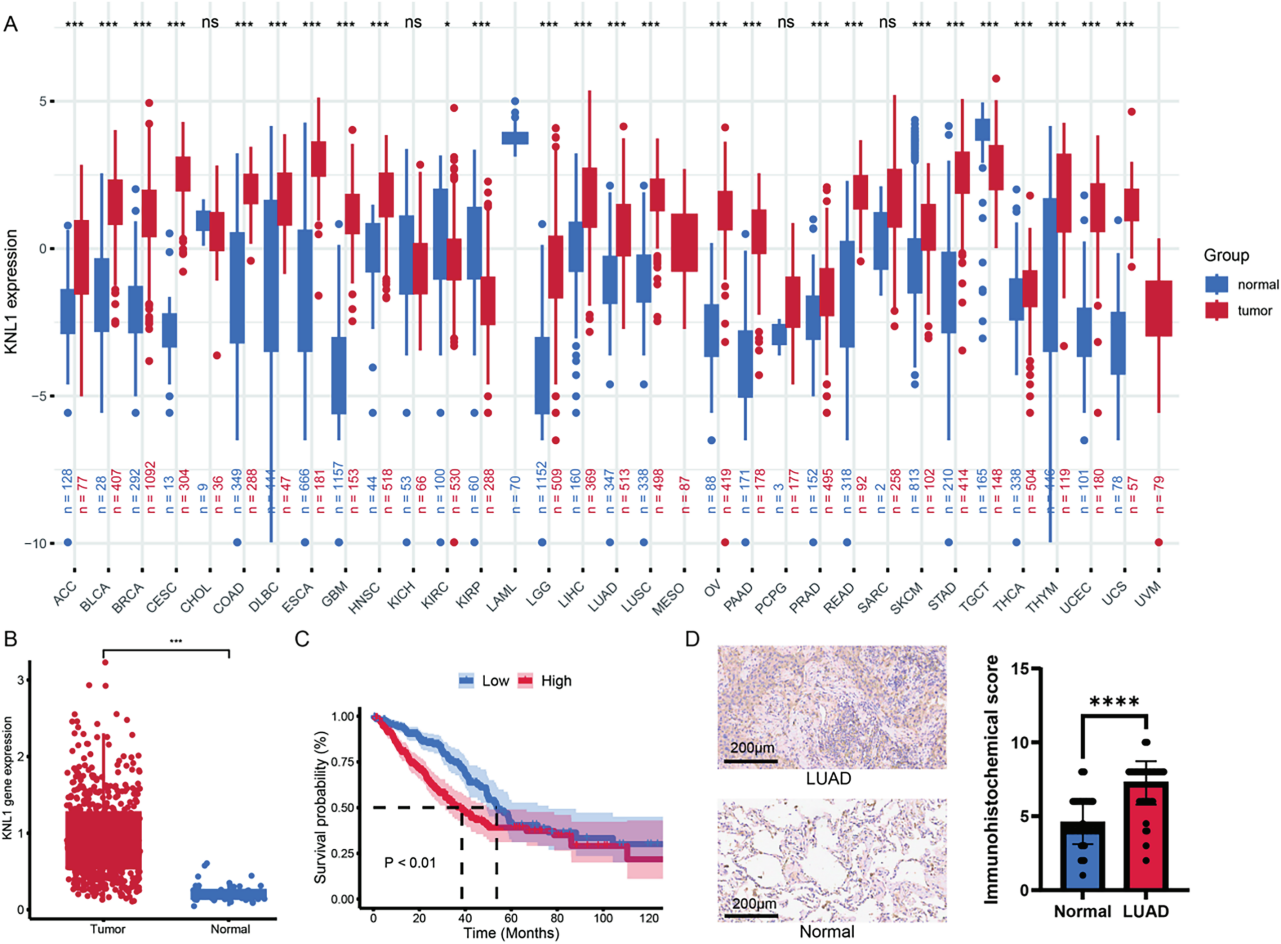
Kinetochore Scaffold 1(KNL1) is significantly up-regulated in lung adenocarcinoma (LUAD) and contributes to poor overall survival. (**A**): Pan-cancer analysis of KNL1 expression using TCGA and GTEx databases, comparing tumor vs. normal tissues across cancer types. (**B**): Differential mRNA expression of KNL1 in TCGA-LUAD cohort. (**C**): Association between KNL1 expression and overall survival in LUAD patients. (**D**): KNL1 immunohistochemical validation in 90 LUAD tissue microarrays from Nanhua University First Affiliated Hospital. *n* = 90. ns. non-significant, *p < 0.05, ***p < 0.001, ****p < 0.0001.

To investigate the potential role of the KNL1 gene in tumorigenesis, this study systematically analyzed the mRNA expression levels of KNL1 across 33 cancer types using bioinformatic methods. Differential analysis revealed significantly elevated KNL1 expression in tumor tissues of 31 cancer types, including LUAD (Fig. 1A,B). Kaplan-Meier survival curves, generated using survival information obtained from TCGA, demonstrated that the median survival time of the high KNL1 expression group was significantly shorter than that of the low expression group (Fig. 1C). Immunohistochemical (IHC) scoring was used to quantify KNL1 expression differences in clinical specimens, which showed significantly higher KNL1 staining intensity in LUAD tissues (Fig. 1D). These results demonstrate that high KNL1 mRNA expression is closely associated with poor prognosis in LUAD patients based on TCGA data, while KNL1 protein is consistently overexpressed in tumor tissues. Together, these findings suggest that KNL1 may have potential as a prognostic indicator.

### 3.2 Role of KNL1 in Lung Adenocarcino Mamigration

#### 3.2.1 Construction of Stable Cell Lines

**Figure 2:**
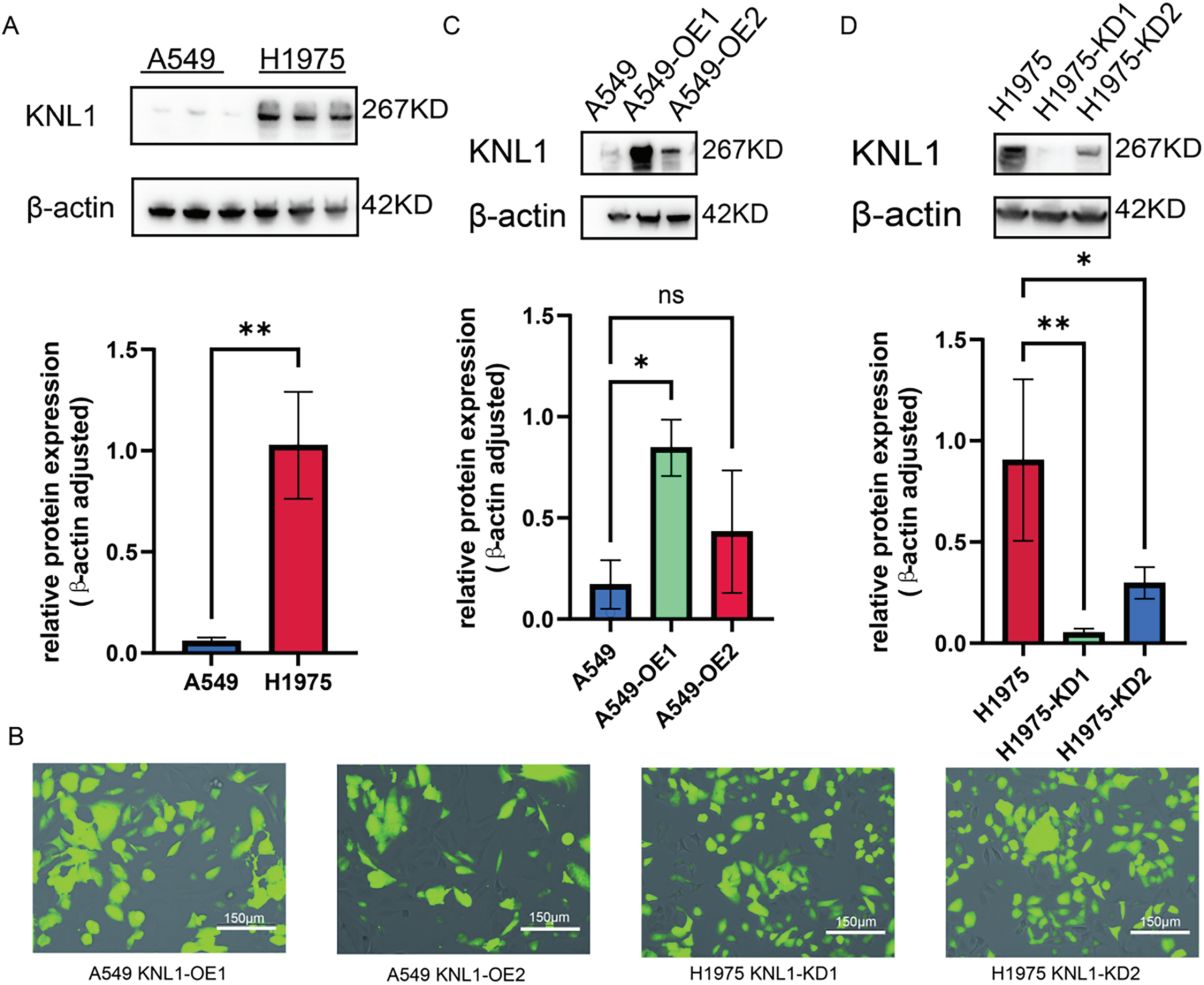
Generation of KNL1-modified stable cell lines. (**A**): KNL1 expression in A549 and H1975 cells, detected by Western blotting. (**B**): Lentiviral reporter fluorescence intensity after transfection. (**C**): KNL1 overexpression in A549 cells using two lentiviruses, detected by Western blotting. (**D**): KNL1 knockdown in H1975 cells using two lentiviruses, detected by Western blotting. *n* = 3, ns. non-significant. *p < 0.05, **p < 0.01.

To investigate the expression regulation and potential functions of the KNL1 protein in different lung cancer cell lines, Western Blot was used to detect KNL1 levels in A549 and H1975 cells. KNL1 protein expression was significantly higher in the H1975 cell line compared to A549 (Fig. 2A). Fluorescence microscopy was performed at 48 h after transfection to monitor reporter gene expression (Fig. 2B). As shown in Fig. 2C,D, lentivirus successfully infected A549 and H1975 cells. Overexpression and knockdown efficiency were confirmed by WB, resulting in the acquisition of stable cell lines.

#### 3.2.2 KNL1 Promotes Lung Adenocarcinoma Migration

**Figure 3:**
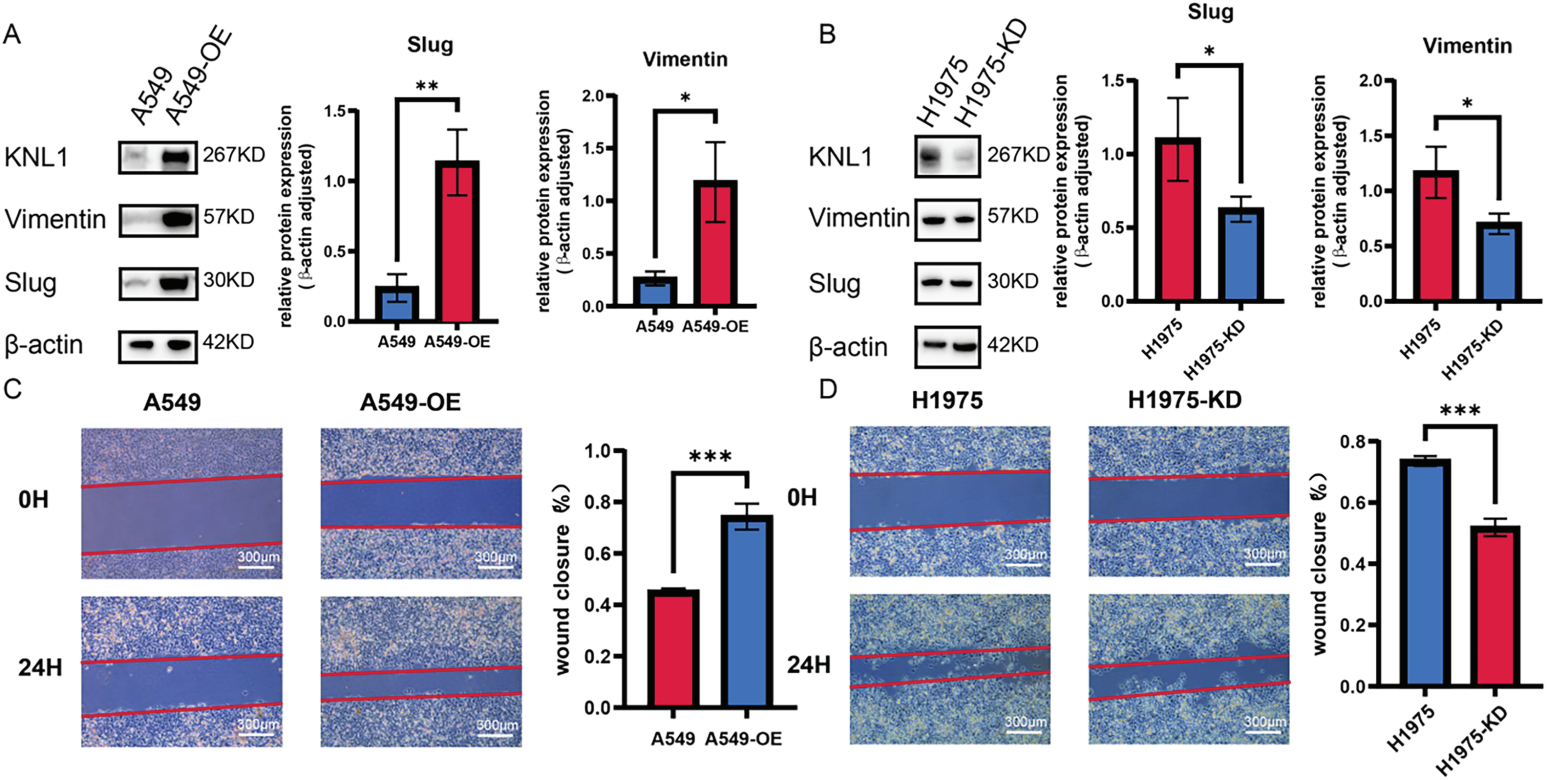
KNL1 promotes lung adenocarcinoma migration. (**A**): Slug and Vimentin expression after KNL1 overexpression by Western blotting. (**B**): Slug and Vimentin expression after KNL1 knockdown by Western blotting. (**C**): Cell migration after KNL1 overexpression by scratch assay. (**D**): Cell migration after KNL1 knockdown by scratch assay. *n* = 3, *p < 0.05, **p < 0.01, ***p < 0.001.

WB results showed that the expression of the EMT transcription factor Slug and the mesenchymal marker Vimentin was significantly upregulated in A549-OE-KNL1 cells (Fig. 3A), while it was significantly downregulated in H1975-KD-KNL1 cells (Fig. 3B). The wound healing assay revealed that the percentage of wound closure at 24 h was significantly higher in the A549-OE-KNL1 group compared to the control group (Fig. 3C), and significantly lower in the H1975-KD-KNL1 group (Fig. 3D). These results indicate that differential KNL1 expression influences the malignant phenotypes of lung cancer cells.

### 3.3 KNL1 Inhibits RSL3-Induced Ferroptosis in Lung Adenocarcinoma Cells

**Figure 4:**
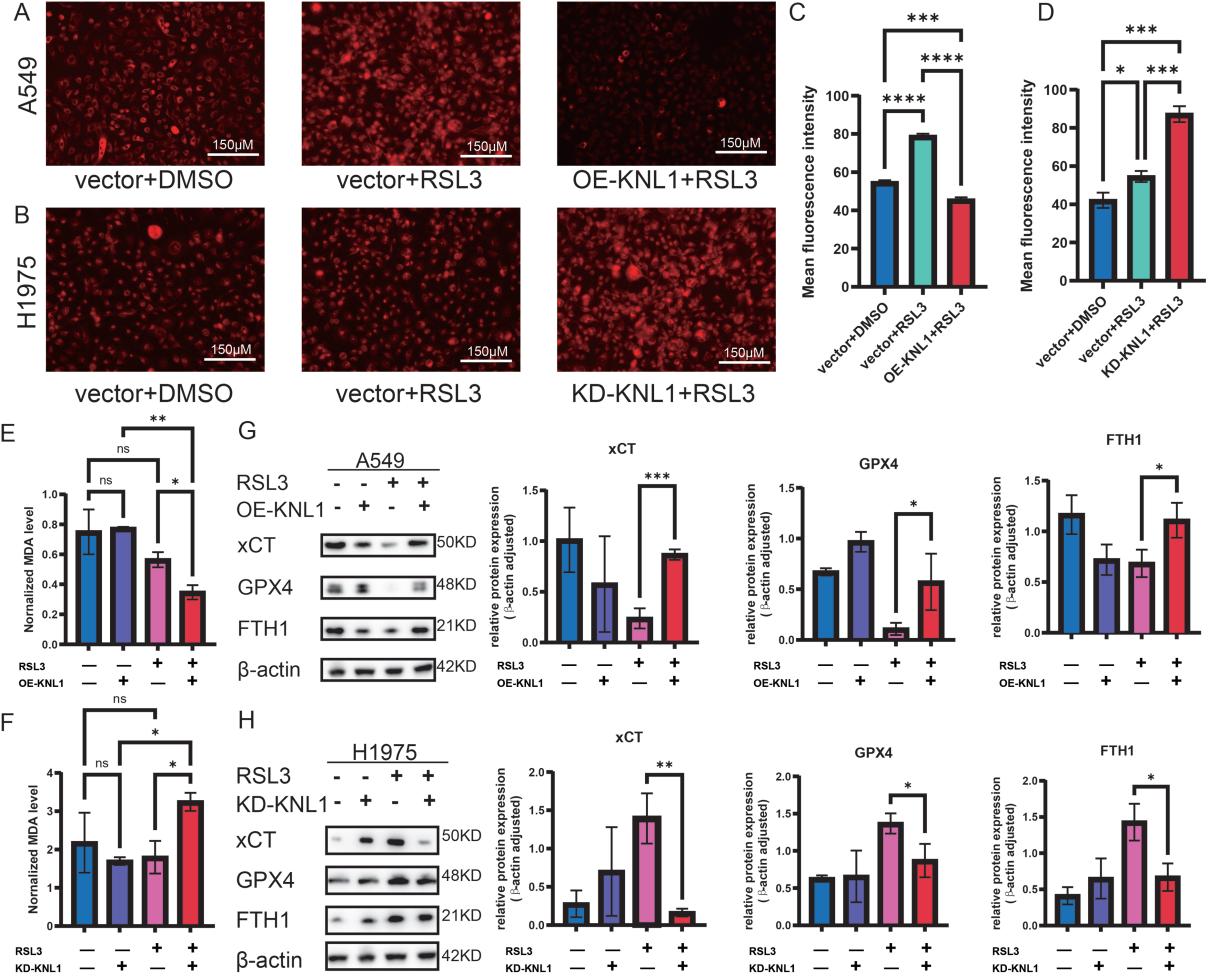
KNL1 inhibits ferroptosis in lung adenocarcinoma under RSL3 treatment. (**A**,**B**): Ferrous iron levels by fluorescent probe. (**C**,**D**): Quantification of mean fluorescence intensity of ferrous iron. (**E**,**F**): Intracellular MDA content. (**G**,**H**): xCT, GPX4, and FTH1 expression by Western blotting. n = 3, ns. non-significant. *p < 0.05, **p < 0.01, ***p < 0.001, ****p < 0.0001.

To explore the effect of KNL1 on RSL3-induced ferroptosis in lung adenocarcinoma cells, A549 and its overexpression cell line were treated with 500 nm RSL3 for 48 h, while H1975 and its knockdown cell line were treated with 300 nm RSL3 for 48 h [17]. As shown in Fig. 4A,B, Fe^2+^ fluorescence was markedly reduced in A549-OE-KNL1 cells and increased in H1975-KD-KNL1 cells after RSL3 treatment. Quantification of fluorescence intensity revealed significant changes in both cell lines (Fig. 4C,D). Meanwhile, intracellular MDA levels were decreased in A549-OE-KNL1 cells but increased in H1975-KD-KNL1 cells (Fig. 4E,F). These changes were associated with altered expression of ferroptosis-related proteins: xCT, GPX4, and FTH1 were upregulated in A549-OE-KNL1 cells (Fig. 4G) and downregulated in H1975-KD-KNL1 cells (Fig. 4H), indicating that KNL1 inhibits RSL3-induced ferroptosis in lung adenocarcinoma cells.

### 3.4 KNL1 Regulates the AMPK-mTOR Signaling Pathway to Inhibit RSL3-Induced Ferroptosis in Lung Adenocarcinoma Cells

**Figure 5:**
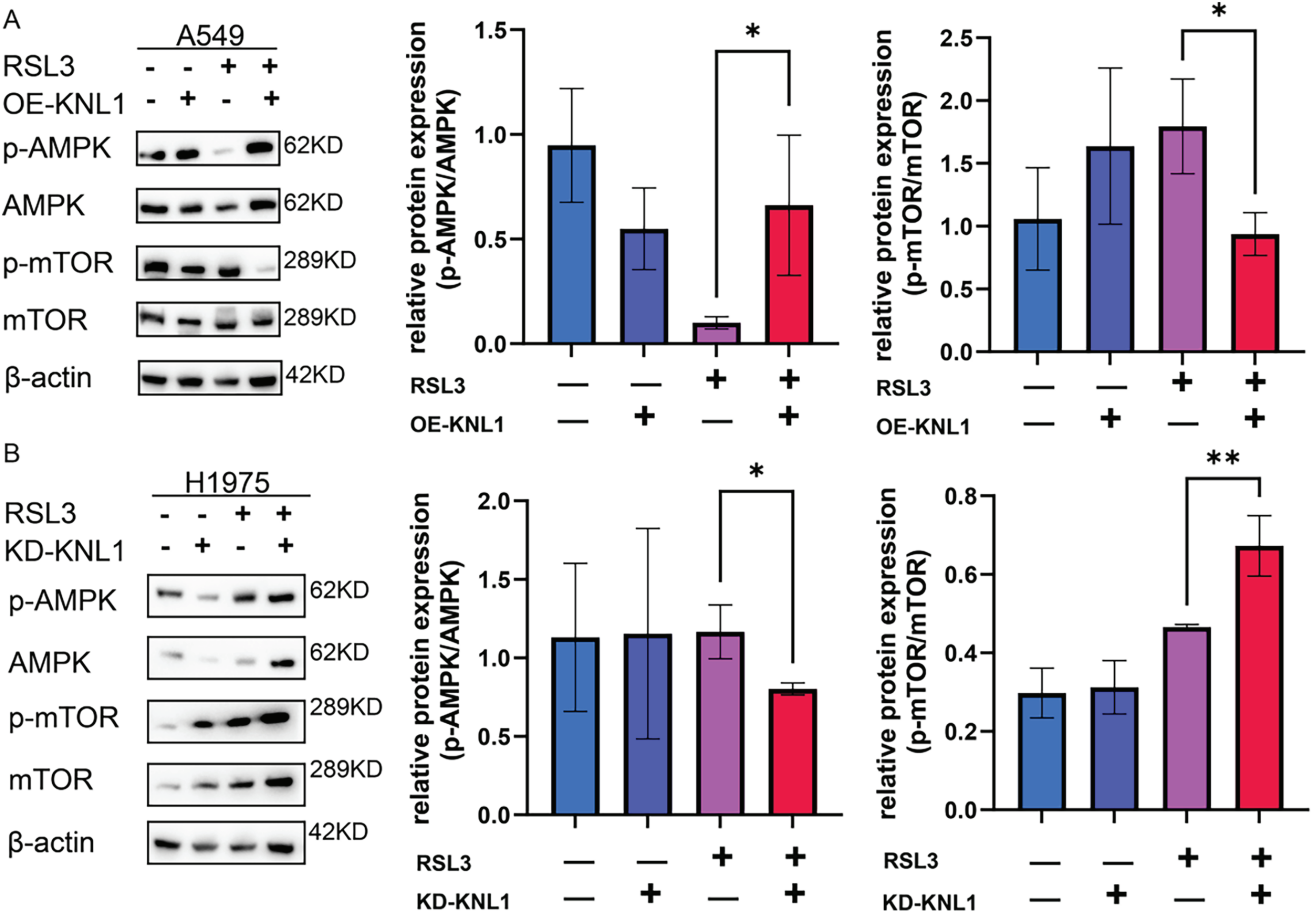
KNL1 modulate AMPK-mTOR signaling by influencing phosphorylation levels. (**A**): Western blot of p-AMPK, AMPK, p-mTOR, and mTOR in A549 cells with KNL1 overexpression or treated with 500 nm RSL3 for 48 h. (**B**): Western blotting of p-AMPK, AMPK, p-mTOR, and mTOR in H1975 cells with KNL1 knockdown or treated with 300 nm RSL3 for 48 h. n = 3, *p < 0.05, **p < 0.01.

To elucidate whether KNL1 regulates ferroptosis through the AMPK-mTOR signaling axis, we analyzed the expression of key proteins in the AMPK-mTOR signaling pathway by Western blot in RSL3-treated cells: including A549-OE-KNL1 cells and H1975-KD-KNL1 cells. The results showed that in RSL3-treated A549 cells, overexpression of KNL1 significantly increased the p-AMPK/AMPK ratio and decreased the p-mTOR/mTOR ratio (Fig. 5A). Similarly, in RSL3-treated H1975 cells, knockdown of KNL1 significantly decreased the p-AMPK/AMPK ratio and increased the p-mTOR/mTOR ratio (Fig. 5B). This suggests that KNL1 may influence the ferroptosis process by regulating the phosphorylation levels of AMPK and mTOR.

## 4 Discussion

Metastasis in LUAD poses a major challenge in clinical management, as distant metastasis often occurs at initial diagnosis, leading to advanced-stage disease with limited sensitivity to radiotherapy and chemotherapy [18]. This results in suboptimal treatment outcomes and a high risk of recurrence and progression. Consequently, precise prognostic assessment and efficacy prediction are urgently needed to guide individualized therapy for LUAD. Current research in LUAD focuses on the role of dysregulated cell death pathways in tumor invasion. Ferroptosis, an iron-dependent form of programmed cell death driven by lipid peroxidation, has emerged as a critical therapeutic target due to its potential to eliminate cancer cells [19,20]. However, it remains unclear how ferroptosis-suppressing regulators in LUAD are linked to metastatic phenotypes. Although the AMPK-mTOR pathway is involved in metabolic and stress responses, its role in ferroptosis remains controversial: AMPK activation may inhibit ferroptosis by suppressing ACC1-mediated lipid synthesis, while mTOR inhibition may downregulate SLC7A11 to promote ferroptosis or enhance monounsaturated fatty acid (MUFA) synthesis via SREBP1/SCD1 to confer resistance [21].

KNL1 is a core kinetochore protein that regulates the spindle assembly checkpoint during mitosis, ensuring proper chromosome-microtubule attachment before anaphase onset [22]. In this study, KNL1 was found to be overexpressed in LUAD at both the mRNA and protein levels, and elevated KNL1 mRNA expression was significantly associated with shortened overall survival in the TCGA cohort, consistent with its reported role as an oncogenic risk factor [23]. While prior research has focused on the relationship between KNL1 and tumor development and clinical prognosis, the regulatory mechanisms remain poorly understood. Our results demonstrate that KNL1 overexpression promotes EMT by upregulating Slug and vimentin and enhancing cell migration, whereas KNL1 knockdown reverses these phenotypes. This expands the known biological functions of KNL1, positioning it as a metastasis-promoting factor. Furthermore, this study shows that KNL1 overexpression reduces RSL3-induced Fe^2+^ accumulation and lipid peroxidation while upregulating xCT, GPX4, and FTH1. Although previous studies have indicated that KNL1 deficiency triggers p53-dependent apoptosis through chromosome missegregation [24]. Our findings further extend the regulatory role of KNL1 in cell death. In summary, our findings suggest that KNL1 is associated with EMT-related migration and resistance to ferroptosis in LUAD cells, implicating it as a potential regulator of tumor cell plasticity and stress adaptation. Further studies are needed to determine whether these associations reflect a functional role in disease progression or therapeutic response.

The AMPK-mTOR signaling pathway, a central regulator of cellular energy metabolism and growth, plays a complex and critical role in tumor initiation, progression, and therapy resistance [25,26]. Some studies have shown that inactivation of liver kinase B1 impedes AMPK phosphorylation, leads to sustained mTOR activation, and upregulates GPX4 expression, thereby conferring ferroptosis resistance [27]. In contrast, our study found that elevated KNL1 expression is associated with increased AMPK phosphorylation and decreased mTOR phosphorylation in LUAD cells under ferroptotic stress. The AMPK-mTOR pathway exhibits context-dependent roles in ferroptosis, as highlighted in prior studies, and discrepancies across reports may stem from differences in tumor molecular subtypes, cellular models, or experimental conditions [28–31]. Thus, identifying robust biomarkers for patient stratification in ferroptosis-targeted therapies remains a critical goal for future investigation. While our data suggest a potential link between KNL1 and ferroptosis regulation through the AMPK–mTOR axis, it should be emphasized that direct mechanistic evidence is lacking. Therefore, future studies employing genetic or pharmacological modulation of AMPK–mTOR signaling are needed to determine whether KNL1 exerts its anti-ferroptotic effects directly through this axis. Nevertheless, KNL1 warrants further evaluation as a candidate biomarker for stratifying patients who may benefit from ferroptosis-inducing therapies.

The present study demonstrates that high KNL1 expression is associated with reduced sensitivity to RSL3-induced ferroptosis in LUAD cells, suggesting a potential role for KNL1 in promoting ferroptosis resistance.

However, this study has limitations, as our experimental approach was restricted to bioinformatics analyses and *in vitro* cell culture models, without rescue experiments to confirm causality or *in vivo* validation in animal models to assess physiological relevance. Consequently, whether KNL1 could serve as a therapeutic target requires further validation in preclinical models, particularly through rescue experiments that restore KNL1 expression following knockdown and *in vivo* studies examining metastasis and therapeutic response in immunocompromised mouse models.

Critically, no small-molecule inhibitors, degraders, or RNAi reagents specifically targeting KNL1 are currently available. This is consistent with its scaffolding function and lack of enzymatic activity, which render it a challenging target for conventional drug development. While our data suggest that KNL1 expression correlates with resistance to RSL3-induced ferroptosis, direct evidence linking KNL1 suppression to enhanced therapeutic response is lacking due to the absence of selective pharmacological or genetic tools. Future studies employing inducible knockdown systems or emerging modalities such as PROTACs or antisense oligonucleotides—should they become feasible for KNL1—could help clarify its functional role in LUAD cell survival. At present, however, KNL1 remains a biologically interesting but not yet druggable node in the context of ferroptosis regulation..

## 5 Conclusion

In conclusion, this study demonstrates that KNL1 overexpression is associated with resistance to RSL3-induced ferroptosis, mesenchymal marker expression, and AMPK-mTOR signaling alterations in LUAD cells, suggesting its potential involvement in cellular plasticity and stress adaptation pathways. These observations contribute to a better understanding of KNL1’s potential role in LUAD and may inform future exploration of ferroptosis-targeted therapeutic approaches.

## Data Availability

The data that support the findings of this study are openly available in UCSC Xena at https://xenabrowser.net/datapages/ (TCGA-LUAD and additional cancer cohorts) and the GTEx Portal at https://www.gtexportal.org/home/ (normal tissue gene expression), and are also available on request from the corresponding author.
